# The Suppressive Role and Aberrent Promoter Methylation of BTG3 in the Progression of Hepatocellular Carcinoma

**DOI:** 10.1371/journal.pone.0077473

**Published:** 2013-10-17

**Authors:** Zhenbing Lv, Huichun Zou, Kaiwen Peng, Jianmei Wang, Yi Ding, Yuling Li, Xiaoli Ren, Feifei Wang, Rui Chang, Li Liang, Yanqing Ding

**Affiliations:** 1 Department of Pathology, Southern Medical University, Guangzhou, Guangdong Province, People’s Republic of China; 2 Department of Pathology, School of Basic Medical Sciences, Southern Medical University, Guangzhou, Guangdong Province, People’s Republic of China; 3 Department of General Surgery, Nanchong Central Hospital, Nanchong City, Sichuan Province, People’s Republic of China; 4 Graduate School, Southern Medical University, Guangzhou City, Guangdong Province, People’s Republic of China; 5 Department of Radiotherapy, Nanfang Hospital, Southern Medical University, Guangzhou City, Guangdong, People’s Republic of China; 6 Second School of Clinical Medicine, Southern Medical University, Guangzhou City, Guangdong Province, People’s Republic of China; Sapporo Medical University, Japan

## Abstract

**Background:**

BTG3 (B-cell translocation gene 3) has been identified as a tumor suppressor and hypermethylation contributes to its down-regulation in some tumors, but its role in hepatocellular carcinoma (HCC) remain unknown. This study aimed to detect the expression and methylation status of BTG3 in HCC cell lines or tissues, and determine its function in HCC progression.

**Methodology:**

The expression of BTG3 was detected in HCC cell lines and HCC tissue by real-time RT-PCR, Western blot or immunohistochemistry. The promoter methylation status of BTG3 was measured by using methylation-specific PCR in HCC cell lines. A series of assays were performed to evaluate the effect of BTG3 on proliferation, invasion and cell cycle transition *in* *vitro.*

**Results:**

BTG3 expression was lower in HCC cell lines than in hepatocyte cell line LO2 (P<0.05). BTG3 was also down-regulated in HCC tissues. Its expression was positively correlated with differentiation and distant metastasis (P<0.05). Patients with lower BTG3 expression had shorter overall survival time (P=0.029). DNA methylation directed repression of BTG3 mRNA expression in HCC cell lines. BTG3 suppressed proliferation, invasion and induces G1/S cycle arrest of HCC cells *in* *vitro.*

**Conclusion:**

Down-regulation of BTG3 due to the promoter hypermethylation is closely associated with proliferation, invasion and cell cycle arrest of HCC cells. It may be a novel prognostic biomarker for HCC patients.

## Introduction

Primary hepatocellular carcinoma (HCC) is the sixth most common cancer worldwide in terms of numbers of cases of 626,000, and the third most common cause of death from cancer (598,000 deaths annually)[1]. The poor prognosis of HCC-patients often connected with late diagnosis and limited therapeutic strategies[2]. The molecular pathogenesis underlying HCC in humans remains poorly understood. Thus, finding some novel molecular markers and studying their functions in HCC may be helpful to understand this neoplasm and adopt new therapeutic options.

B-cell translocation gene 3 (BTG3) belongs to an anti-proliferative B-cell translocation gene/Transducer of ErbB2 (BTG/Tob) gene family, which also includes BTG1, BTG2/TIS21/PC3, Tob, Tob2 and PC3b in human cells[3]. These proteins all contain two short conserved domains in their N-terminal part (box A and box B), separated by a spacer sequence of 20-25 non-conserved amino acids[3–5]. So far, Evidences for the family not only inhibiting cellular proliferation and differentiation, but also involving in the regulation of tumorigenic progression have been reported[6]. Overexpression of BTG4 can suppress colony formation in colorectal cancer cells and its expression is frequently down-regulated in primary gastric cancers[7,8]. PC3/BTG2 mRNA is highly expressed in HCC cells and its expression is related to the degree of cell differentiation[9]. TOB plays an important role in the suppression of breast cancer tumorigenesis[10]. 

Recent evidence demonstrates that BTG3 plays a suppressive role in cancer progression. Loss of BTG3 expression correlates with the development of lung adenocarcinoma, oral squamous cell cancer or prostate cancer[11–13]. Aberrant epigenetic regulation of BTG3 promoter, such as by DNA hypermethylation and/or histone modification is observed in several human cancers[14-17]. Till now, only two recent papers have discussed the function of BTG3 in tumor[13,18]. BTG3 is a downstream target of p53 and also binds and inhibits E2F1. It connects functionally those two major growth-regulatory pathways[18]. BTG3 triggers acute cellular senescence via the ERK-JMJD3-p16 signaling axis[13]. 

However, the expression pattern and functions of BTG3 in HCC remain unknown. In this study, we detected the expression and methylation status of BTG3 in HCC cell lines and clinical samples, and determined its prognostic value. Then we examined the effect of BTG3 on HCC cell proliferation, cell cycle and invasion *in vitro*. 

## Materials and Methods

### DNA methylation analysis

Genomic DNAs of LO2, HepG2 and 97H cells[19-21] were obtained using GeneJET Genomic DNA purification Kit (Thermo, Salt Lake city, USA) and then bisulfite-modified using the Epitect Plus DNA Bisulfite Kit (Qiagen, Valencia, Calif). The CpG island of BTG3 gene was predicted online (http://www.urogene.org/methprimer/index1.html). The primers used in bisulfite-specific PCR (BSP) detection were designed as following (F: 5'-AGG TTG AGA TAG TTG AAA GGA TTA AGTT-3'; R: 5'-CAA AAA AAA AAA AAA ACA ATA ACC CAA AAA AAA ATT AA-3'). The PCR reaction was performed at 95°C for 5min, then 30 cycles of 95°C for 30sec, 56°C for 30sec, 72°C for 30sec, followed by an extra extension at 72°C for 5min. The BSP products were confirmed by electrophoresis on a 2% agarose gel. Finally, they were cloned into a T-vector (TaKaRa, Japan), and sequenced (Taihegene Biotechnology Co Ltd, Beijing, China).

### Cell proliferation assay

1×10^3^ cells were seeded into 96-well plates. The number of viable cells was determined by cell counting kit-8 (CCK-8) (Dojindo, Kumamoto, Japan) for 6 days. Briefly, 10 μL CCK-8 solution was added, and absorbance at 490 nm was measured after 2 h of incubation at 37°C. Each cell group was plated in 3 duplicate wells.

### Cell-cycle analysis

About 1×10^6^ cells were trypsinized, washed twice with PBS, and fixed in 70% ice-cold ethanol for 1 h. The samples were then centrifuged by removing the ethanol and exposing to 100 mg/mL RNaseA (Sigma, USA) for 30 min at 37°C. Cellular DNA was stained with propidium iodide. Cell-cycle distributions were determined using flow cytometry.

### In vitro invasion assay

The invasion Boyden chambers (BD Biosciences, Foster city, USA) were rehydrated with RPMI 1640 (serum-free) for 2h at 37°C. RPMI 1640 with 100 ml/L fetal bovine serum was added to the lower compartment as the chemotactic factor. Then 1.5×10^5^ tumor cells in serum-free RPMI 1640 were added to the upper compartment of the chamber. Each cell group was plated in 3 duplicate wells. After incubation for 48 hr, the noninvasive cells were removed with a cotton swab. Cells that had migrated through the membrane and stuck to the lower surface of the membrane were fixed with methanol and stained with hematoxylin. Finally, the cells in lower compartment of the chamber that had invaded the lower sides of the membrane were counted under a light microscope in 5 random visual fields (200×).

Materials of HCC cell lines and clinical tissue specimens, immunohistochemistry, real-time RT-PCR, Western blotting, construction of plasmids and transfection, statistical analysis were seen in Supplementary Materials and Methods. 

## Results

### BTG3 expression in human HCC tissues

**Figure 1:**
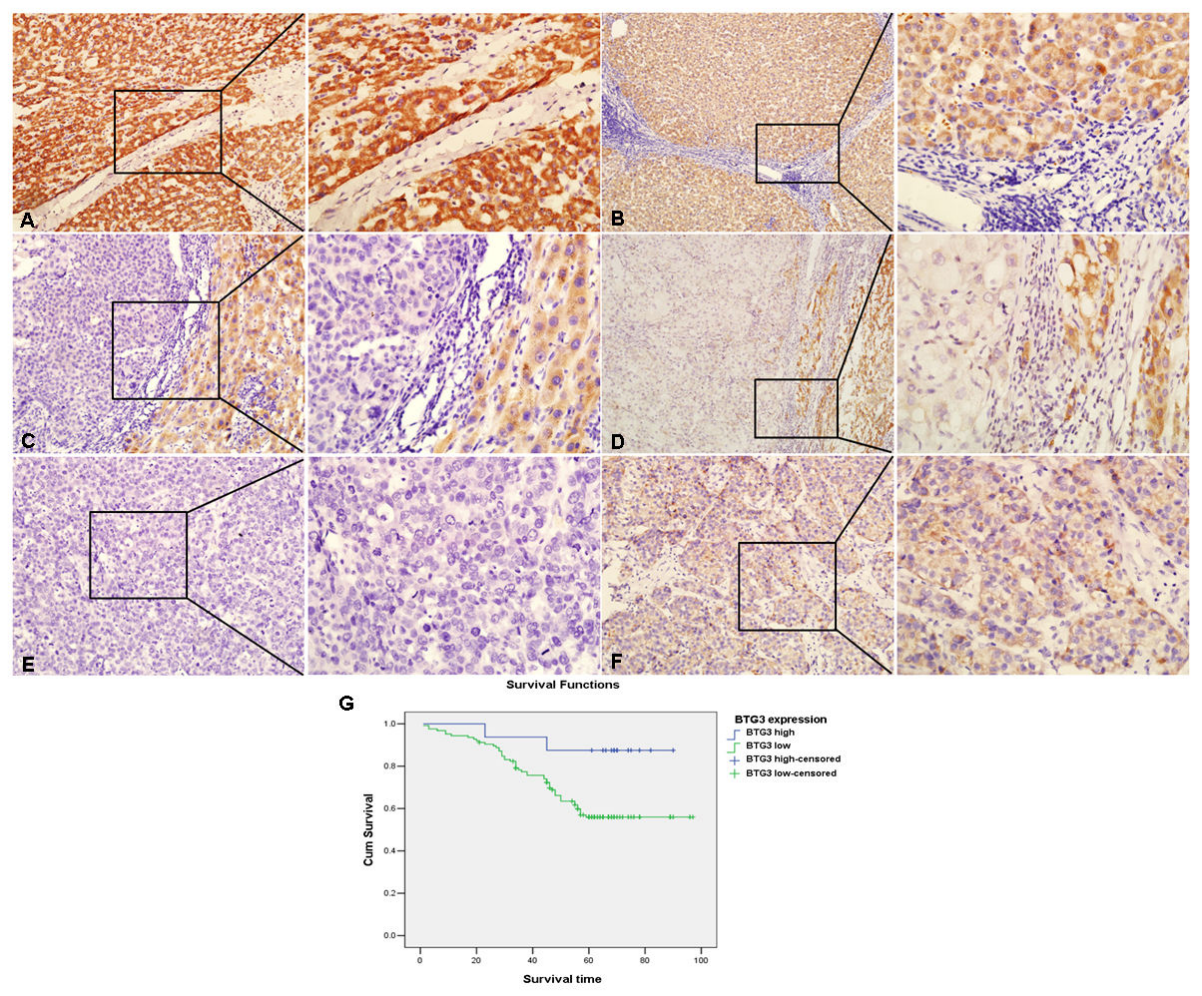
BTG3 is down-regulated in clinical paraffin-embedded HCC tissues and a prognostic factor for poor overall survival in HCC patients. (A) Strong positive expression of BTG3 in adjacent normal livers (×200, ×400). (B) Positive expression of BTG3 in the cirrhotic livers (×100, ×400). (C) Negative expression of BTG3 in HCC tissues compared with strong positive expression in adjacent livers (×200, ×400). (D) Week expression of BTG3 in HCC tissues while strong positive expression in adjacent livers (×200, ×400). (E) Negative expression of BTG3 in HCC tissues with low differentiation (×200, ×400). (F) Weak expression of BTG3 in HCC tissues with high differentiation (×200, ×400). (G) Kaplan-Meier survival analysis of primary HCC patients with high and low BTG3 expressions.

To explore the expression pattern of BTG3 and its clinicopathologic features in HCC patients, we analyzed BTG3 protein expression in 141 cases of paraffin HCC specimens using IHC. Strong staining for BTG3 protein was frequently observed in the cytoplasm of adjacent normal liver cells (Figure 1A) and week positive signals were mainly seen in cirrhotic livers (Figure 1B). On contrast, negative or very week BTG3 staining was observed in HCC tissues (Figure 1E, 1F). Down-regulation of BTG3 in HCC tissues could be appreciated in sections containing both normal and cancerous tissues (Figure 1C, 1D). The expression of BTG3 was obviously lower in HCC tissues than non-cancerous liver tissues (P<0.001, Table S1). No obvious difference of BTG3 expression was seen between HCC tissues with cirrhosis and those without cirrhosis (P<0.001, Table S1). BTG3 expression was correlated strongly with differentiation (P=0.038) and distant metastasis (P=0.043, Table 1). These results indicate that down-regulation of BTG3 may be associated with the progression of HCC.

**Table 1:** Relationship between BTG3 expression and clinicopathologic features of HCC patients.

**Features**	**Total Number**	**High expression**	**Low expression**	**P**	**λ^2^**
All case	141	16	125		
Age				0.865	0.033
<55		10	81		
>=55		6	44		
Gender				0.378	0.309
Male		13	108		
Female		3	17		
Tumor size				0.092	2.835
<5cm		11	58		
>=5cm		5	72		
Differentiation				**0.038**	6.538
Well		1	25		
Moderate		8	78		
Poor		7	22		
Cirrhosis				0.616	0.251
N		7	63		
Y		9	62		
Distant Metastasis				**0.043**	3.36
N		14	79		
Y		2	46		
Relapse				0.621	0.244
N		10	70		
Y		6	55		
Portal Vein Thrombosis				0.966	0.002
N					
Y		13	101		
		3	24		
Dissemination				0.092	2.835
N		11	58		
Y		5	67		
HBsAg				0.166	1.914
negative		2	36		
positive		14	89		
Serum AFP				0.276	1.185
>25ng		8	45		
≦25ng		8	80		

### Correlation between BTG3 expression and patients’ survival

The prognostic effect of BTG3 on HCC patients’ overall survival was compared between patients with high and low BTG3 protein levels by Kaplan-Meier curve assessment. We found that low BTG3 protein level was a significant prognostic factor for poor overall survival in HCC patients (P=0.029, Figure 1G). From univariate analysis, the significant prognostic factors were BTG3 expression, portal vein thrombosis, differentiation, distant metastasis, disseminationand relapse (P<0.05, Table S2). Multivariate analysis results showed that differentiation and dissemination might play a role in predicting the overall survival in HCC patients (P<0.05, Table S2). These results show that BTG3 expression is an independent prognostic marker for survival of HCC patients.

### BTG3 expressions in HCC cell lines and fresh HCC tissues

**Figure 2:**
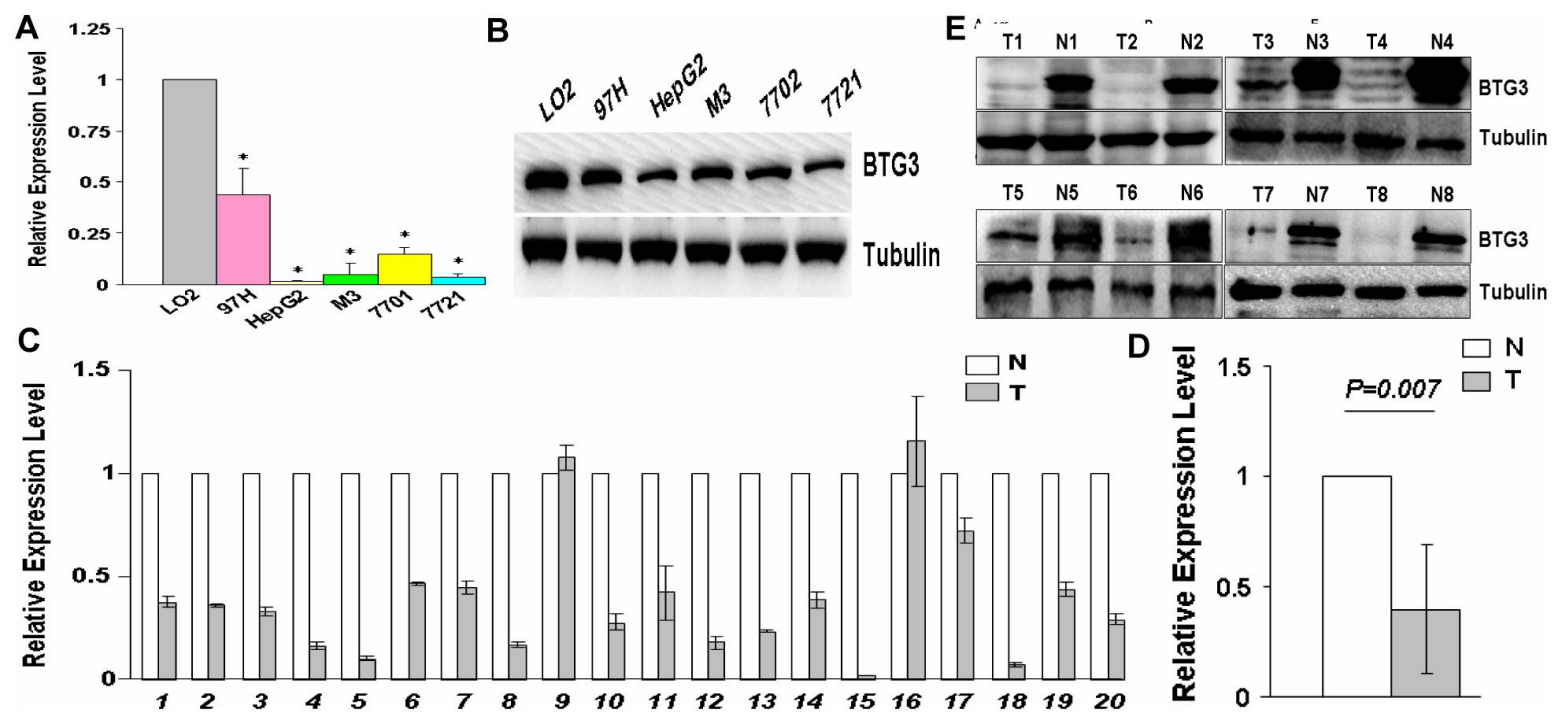
BTG3 is down-regulated in HCC cell lines and fresh HCC tissues. (A) Real-time RT-PCR analysis of BTG3 in six cell lines. The relative mRNA levels with the use of control LO2 were normalized to 1. (B) Western blotting analysis of BTG3 in six cell lines. Tubulin was shown as a control. (C) Real-time PCR analysis of BTG3 expression in 20 paired HCC tissues. The relative mRNA levels with the use of control normal livers were normalized to 1. (D) Real-time PCR analysis of BTG3 expression in all cases of HCC tissues and adjacent normal livers. (E) Western blotting analyses of BTG3 expression in 8 paired HCC tissues. Tubulin was shown as a control. N=normal mucosa and T=tumor.

To further validate the down-regulation of BTG3 in HCC, we examined the expression level of BTG3 in 5 HCC cell lines and 20 matched pairs of fresh HCC tissues by real-time PCR and Western blot. The expression of BTG3 was drastically lower in 5 HCC cell lines than in a hepatocyte cell line LO2 (Figure 2A). Consistently, Western blot analysis demonstrated that BTG3 was also down-regulated in 5 HCC cell lines (Figure 2B). BTG3 was obviously down-regulated in 20 cases of fresh HCC tissues compared to adjacent noncancerous livers by real-time PCR (P<0.001, Figure 2D). Only two HCC tissues exhibited up-regulated expression of BTG3 (Figure 2C). The level of BTG3 protein expression in those fresh HCC tissues by Western blot coincided with that of the mRNA level (Figure 2E). Together, the above data confirm the decreased expression of BTG3 in HCC. 

### Methylation status of BTG3 in HCC cell lines

**Figure 3:**
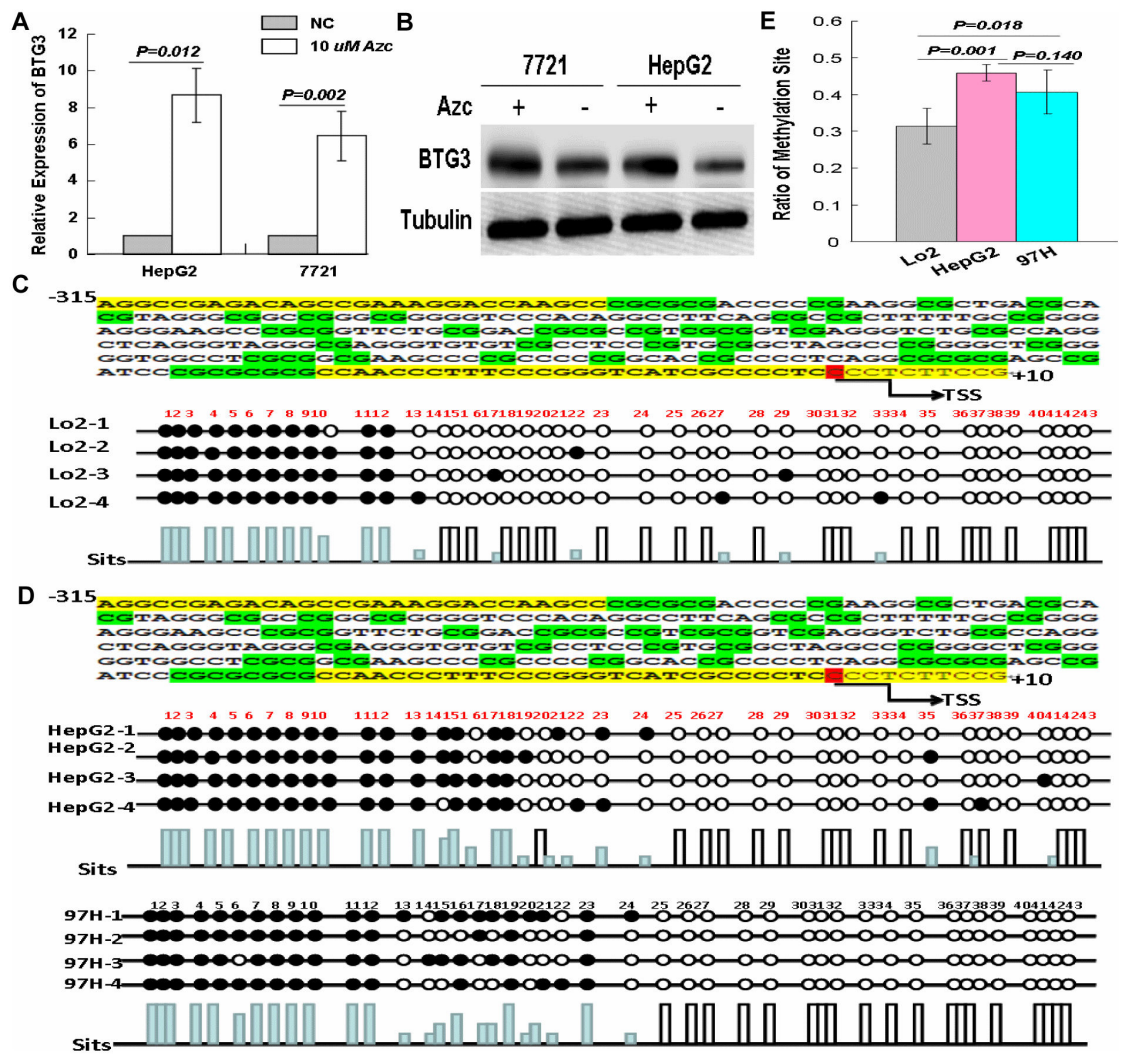
Promoter hypermethylation contributes to the transcriptional silencing of BTG3 in HCC. (A) Real-time PCR analysis of BTG3 expression after 5Aza-C treatment in HepG2 and 7721 cells. The relative mRNA levels with the use of control cells without 5Aza-C treatment were normalized to 1. (B) Western blotting analysis of BTG3 expression in HepG2 and 7721 cells after 5Aza-C treatment. Tubulin was shown as a control. (C) DNA sequencing results of methylation sites in LO2 cells. (D) DNA sequencing results of methylation sites in HepG2 and 97H cells. (E) Ratio of methylation sites of HepG2 or 97H cells was compared with that of LO2 cells.

To check whether transcriptional silencing of BTG3 gene is due to promoter hypermethylation, we used the 5Aza-C treatment in HepG2 and 7721 cells and found that there were increased mRNA and protein levels of BTG3 in 5Aza-C treated samples (P<0.01, Figure 3A, 3B). Next, we analyzed the status of promoter methylation for BTG3 in LO2, two HCC cell lines HepG2 and 97H by bisulfite-modified PCR. DNA sequencing results reveals the promoter of BTG3 gene in HepG2 and 97H cells was hypermethylated in comparison to LO2 cells (P<0.05, Figure 3C-3E). The BTG3 promoter in two HCC cell lines was completely methylated, whereas there was a partial absence of CpG island methylation in LO2 (Figure 3C, 3D). These results indicate that transcriptional silence of BTG3 gene is due to promoter hypermethylation. 

### Effect of BTG3 over-expression on HCC cell proliferation, invasion and cell cycle transition

**Figure 4:**
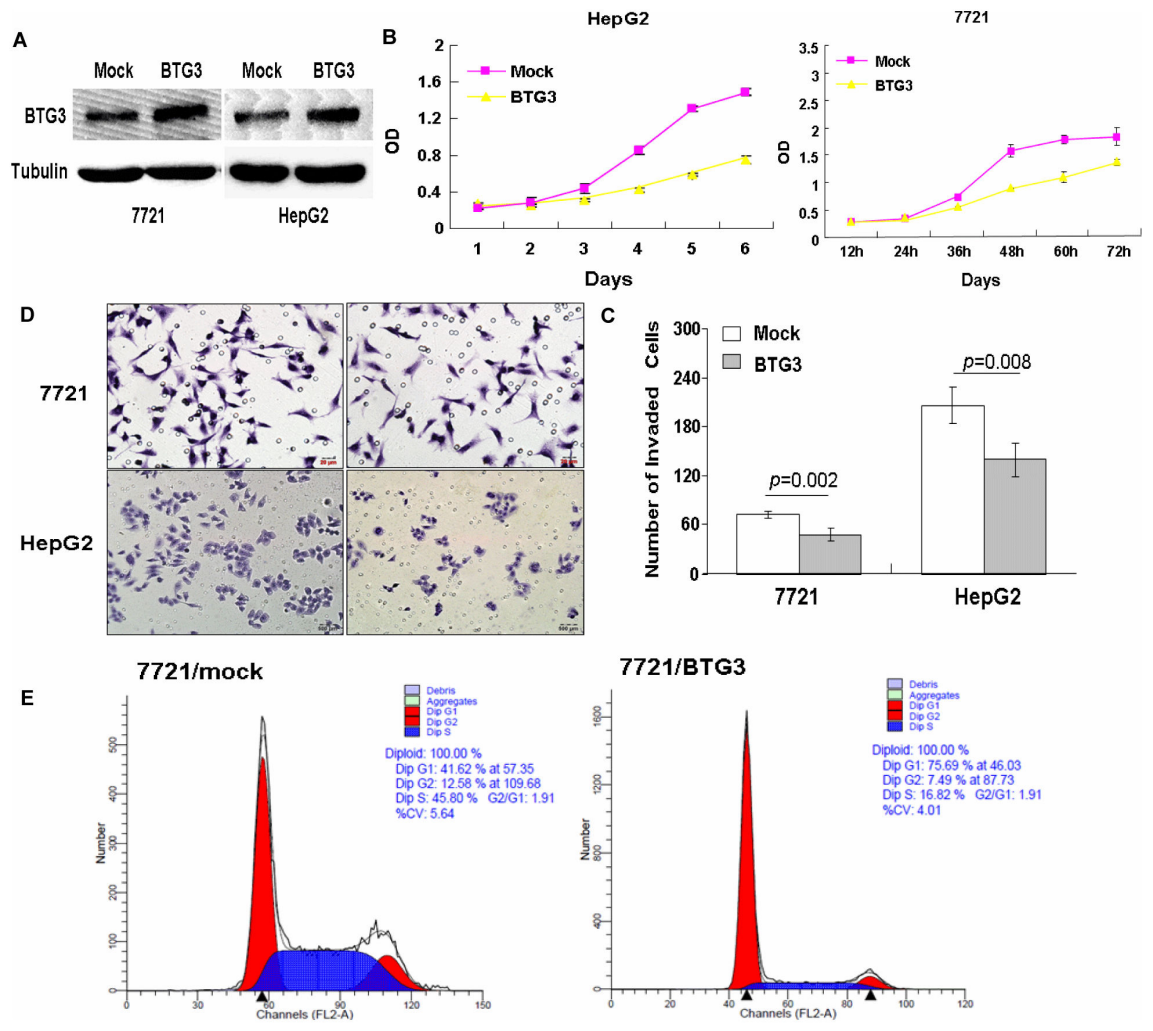
BTG3 inhibits proliferation, invasion and induces G1/S cycle arrest of HCC cells in vitro. (A) BTG3 expression in FBX8 over-expressing 7721 and HepG2 cells by Western blot. Tubulin was shown as a control. (B) Effect of ectopic BTG3 on cell proliferation in vitro by MTT assay. (C) Effect of ectopic BTG3 on cell invasion in vitro by using Boyden chambers. (D) Morphological comparison of BTG3-overexpressing cells and control cells penetrating the artificial basement membrane was shown. (E) Effect of ectopic BTG3 on cell cycle transition in vitro by flow cytometry.

To elucidate the role of BTG3 in the progression of HCC, we performed a series of assays to detect the effect of BTG3 over-expression on cancer cell proliferation, cell cycle transition and invasion *in vitro*. The BTG3 expression vector was constructed and transfected into HepG2 and 7721 cells (Figure 4A). The CCK8 assay showed that forced expression of BTG3 caused a significant decrease of the proliferation rate (P<0.05, Figure 4B). Results of the Boyden chamber assay showed that BTG3-overexpessing cells displayed the marked suppression of invasive ability compared with mock cells (P<0.05, Figure 4C). The number of cells penetrating the artificial basement membrane obviously increased in BTG3-overexpressing cells (Figure 4D). The flow cytometric analysis showed that ectopic BTG3 in 7721 cells caused a significantly increased G1-phase cell population, and a decreased cell population in S-phase (Figure 4E). These data suggest that BTG3 suppresses HCC cell proliferation, invasion and induces G1/S cycle arrest. 

### Effect of BTG3 knockdown on HCC cell proliferation, invasion and cell cycle transition

**Figure 5:**
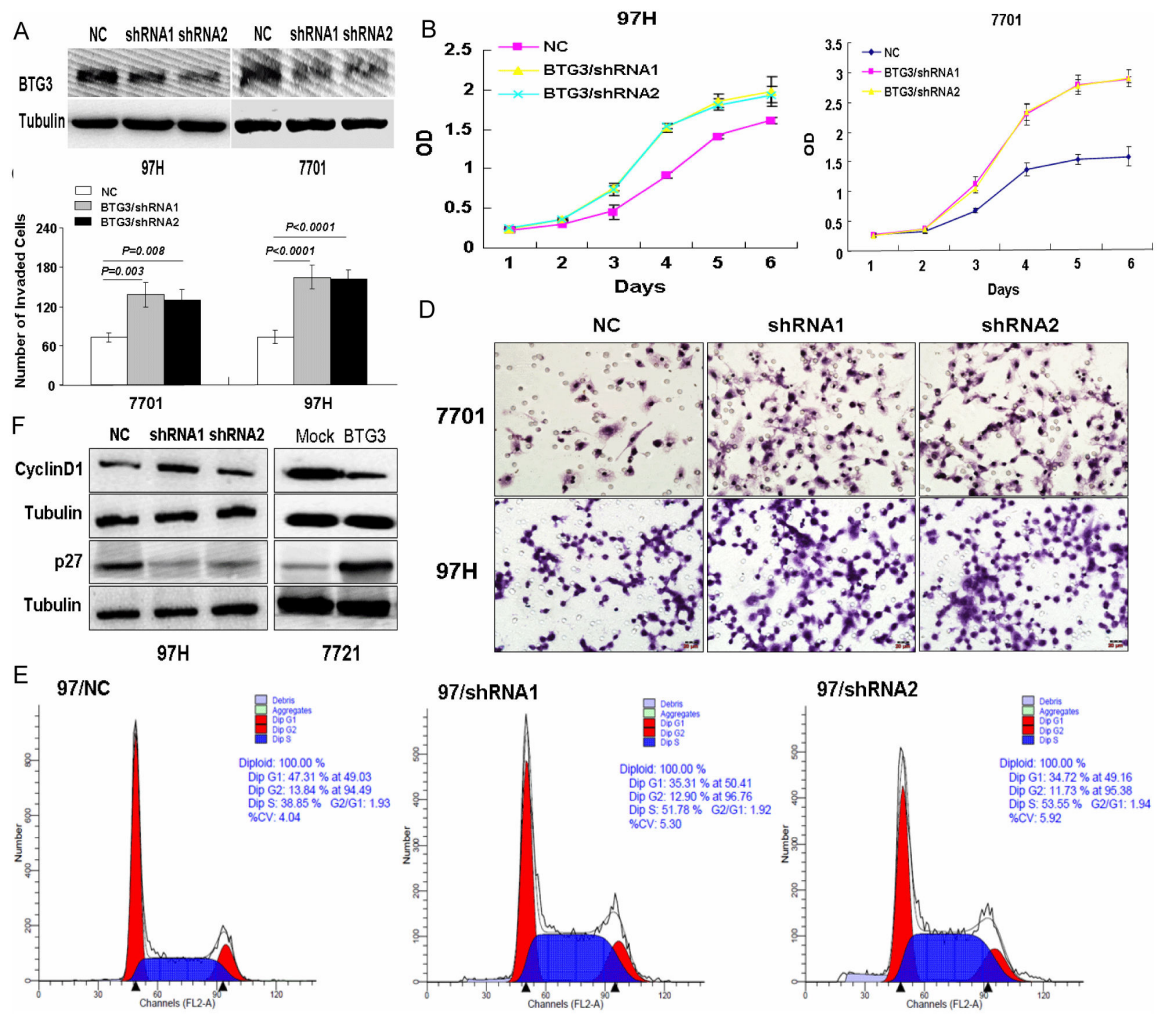
BTG3 knockdown promotes proliferation, invasion and G1/S phase transition of HCC cells in vitro. (A) BTG3 expression in BTG3 depleting 97H and 7701 cells by Western blot. (B) Effect of BTG3 knockdown on cell proliferation in vitro by MTT assay. (C) Effect of BTG3 knockdown on cell invasion in vitro by using Boyden chambers. (D) Morphological comparison of BTG3-depleting cells and control cells penetrating the artificial basement membrane was shown. (E) Effect of BTG3 kncokdown on cell cycle transition in vitro by flow cytometry. (F) Expression levels of Cyclin D1 and p27 in BTG3-overexpressing and depleting cells by Western blot. Tubulin was shown as a control.

We also assessed the effect of BTG3 knockdown on HCC cell proliferation, invasion and cell cycle transition *in vitro*. We constructed BTG3-depleting 97H and 7701 cell models using two BTG3-specific shRNAs (Figure 5A). Results showed that BTG3 depletion resulted in a more dramatic increase of the proliferation rate in 97H and 7701 cells than in control cells (P<0.05, Figure 5B). Knockdown of BTG3 also strongly enhanced the invasive abilities in two cell lines (P>0.05, Figure 5C). The number of invaded cells in 97H/shRNA and 7701/shRNA groups was obviously more than in control groups (Figure 5D). FCM analysis showed that S-phase rate increased markedly in 97H/shRNA cells compared with control cells, and down-regulation of BTG3 promoted G1/S transition of cell cycle (Figure 5E). To reveal the possible mechanisms for BTG3-regulated cell cycle in HCC, we examined the expressions of CyclinD1 and cyclin-dependent kinase inhibitor p27 in 97H/shBTG3 cells and 7721/BTG3 cells. Western blot showed that BTG3 over-expression resulted in up-regulated p27 and down-regulated CyclinD1, while BTG3 knockdown showed the opposite effects (Figure 5F). The above results suggest that knockdown of BTG3 alone is sufficient to promote proliferation, invasion and G1/S phase transition of HCC cells. 

## Discussion

As BTG3 was isolated by low-stringency screening of a cDNA library by using BTG1 and BTG2/TIS21 probes[4], its physiological functions started to be found, such as neuron phylogenesis, the control of muscle cell development and bone formation^[17]^, especially, for the control of cell cycle[5,6,12,13]. Several studies have shown that BTG3 was associated with tumorigenesis. It has been reported as a candidate tumor suppressor gene in several cancers[13-16,18]. However, the expression pattern and functions of BTG3 in the progression of HCC remain elusive.

In this study, we provide evidence that BTG3 plays an important suppressive role in HCC progression especially tumor cell proliferation, invasion and cell cycle progression. First, we detected the expression of BTG3 in a large cohort of clinical paraffin-embedded HCC tissues with long-term follow-up, 20 paired fresh HCC tissues and 5 HCC cell lines. The results showed that BTG3 had a higher probability of being down-regulated in HCC tissues. BTG3 expression was an independent prognostic marker for survival of HCC patients. Moreover, BTG3 expression, portal vein thrombosis, differentiation, distant metastasis, dissemination and relapse were singled out as six marked and independent prognostic factors relative to overall survival. Besides BTG3 expression, the five other factors are well-acknowledged indicators in the progression of HCC[22-26]. Down-regulation of BTG3 was also observed in 20 paired fresh HCC tissues and 5 HCC cell lines compared with the hepatocyte cell line LO2. Many BTG proteins such as BTG4, TOB1, TOB2 are aberrantly expressed and serve as negative regulators in tumorigenesis in various cancers[8,10,27]. Our data clearly demonstrate that the decreased expression of BTG3 is associated with the progression of HCC and an independent prognostic marker for survival of HCC patients. 

Increased DNA methylation of CpG islands in the promoter region of genes is well established as a common epigenetic mechanism for the silencing of tumor suppressor genes in cancer cells[16,28]. Aberrant hypermethylation status of BTG3 promoter was reported in some human cancers[14-18]. In this study, we examined whether transcriptional silencing of BTG3 was due to the promoter hypermethylation. Results showed that down-regulation of BTG3 in two HCC cell lines could be reversed by 5Aza-C treatment, which forms a covalent complex with the active sites of methyltransferase resulting in generalized demethylation[29]. It was expected that promoter of the BTG3 gene in HepG2 and 97H cells was hypermethylated in comparison to LO2 cells. Our data indicate that the promoter hypermethylation contributes to down-regulation of BTG3 in HCC.

The biological meaning of down-regulated BTG3 in HCC cells and tissues remains unclear. In-depth studies are needed to clarify the aberrant roles of BTG3 in HCC progression. Second, a series of relevant functional experiments in vitro, from positive to negative, were performed in our study. Here, we observed that BTG3 could strongly inhibit the proliferative abilities of HCC cell lines. As a member of the anti-proliferative gene family, over-expression of BTG3 also inhibits cell proliferation in breast cancer[16]. The inhibition of cell proliferation by BTG3 is thought to result from its negative regulation of cell cycle[4,14]. Our flow cytometric analysis showed that BTG3 was expressed highly in late G1 phase before the entry of the cells in S phase, while down-regulation of BTG3 promoted G1/S cycle transition of HCC cells. Several reports demonstrate that BTG3 constitutes important negative regulatory mechanism for Src-mediated signaling and it inhibits transcription factor E2F1, suggesting it has a negative regulatory influence consistent with its role to inhibit progression into S-phase, meanwhile, BTG3 was identified as a transcriptional target of p53[14,18]. BTG3 interacts with CHK1, a key effector kinase in the cell cycle checkpoint response, and regulates its phosphorylation and activation[30]. Moreover, MiR-378 promotes cellular transformation, at least in part, by targeting and inhibiting TOB2, which is further elucidated as a candidate tumor suppressor to transcriptionally repress proto-oncogene cyclin D1[31]. Loss of BTG2 in estrogen receptor-positive breast cancer is associated with overexpression of Cyclin D1 protein[32]. Our data showed that up-regulated p27 and down-regulated Cyclin D1 might be responsible for G1/S cycle arrest induced by BTG3 in HCC. BTG3 was reported to be linked with aggressiveness of ovarian carcinoma[33]. Thus, we hypothesized BTG3 might also play a role in HCC by suppressing invasion of HCC cells. Our results showed that BTG3 displayed the marked suppression of invasive abilities of HCC cells *in vitro*. Thus, down-regulation of BTG3 alone is a necessary factor for cell proliferation, cell cycle transition and invasion in HCC cells. 

In summary, our study presents a significant association between down-regulation of BTG3 through hypermethylation and HCC progression. BTG3 inhibits proliferation through inducing cell-cycle arrest and invasion of HCC cells. BTG3 may be a significant prognostic biomarker of HCC progression. A better understanding of the function of BTG3 in HCC progression would provide a valuable marker and novel therapeutic strategy for HCC patients.

## Supporting Information

Table S1 Expression of BTG3 in HCC tissues, cirrhotic liver and adjacent normal liver tissues. Noncancerous liver tissues VS HCC tissues Z= -13.059 P<0.001 (Wilcoxon Signed Ranks Test).
HCC tissues VS Cirrhotic liver Z= - 9.704 P<0.001 (Wilcoxon Signed Ranks Test).
HCC tissues with cirrhosis VS HCC tissues without cirrhosis λ=1.911, P=0.591 (Chi-Square Tests).
(DOC)

Table S2 Univariate and multivariate analyses of individual parameters for correlations with overall Surval rate: Cox proportional hazards model. Abbreviations: HR, Hazard radio; CI, Confidence interval. Statistically significant (p<0.05).
(DOC)

Materials and Methods S1 (DOC)
