# Supplementary material for: The Suppressive Role and Aberrent Promoter Methylation of BTG3 in the Progression of Hepatocellular Carcinoma
Source: PLoS One. 2013 Oct 17;8(10):e77473. doi: 10.1371/journal.pone.0077473 (PMC3798399; doi:10.1371/journal.pone.0077473)
Supplement: Table S1 — Noncancerous liver tissues VS HCC tissues Z= -13.059 P<0.001 (Wilcoxon Signed Ranks Test). HCC tissues VS Cirrhotic liver Z= - 9.704 P<0.001 (Wilcoxon Signed Ranks Test). HCC tissues with cirrhosis VS HCC tissues without cirrhosis λ=1.911, P=0.591 (Chi-Square Tests). (DOC) [file pone.0077473.s001.doc]

| Group | BTG3 expression | | | | Total |
| --- | --- | --- | --- | --- | --- |
|  | - | + | ++ | +++ |  |
| Noncancerous liver tissues | 4 | 15 | 56 | 66 | 141 |
| Cirrhotic liver | 1 | 7 | 11 | 28 | 47 |
| Adjacent normal liver | 0 | 8 | 19 | 67 | 94 |
| HCC tissues | 95 | 30 | 15 | 1 | 141 |
| HCC tissues with cirrhosis | 4649 | 1713 | 8 | 1 | 72 |
| HCC tissues without cirrhosis |  |  | 7 | 0 | 69 |
