# Supplementary material for: The Suppressive Role and Aberrent Promoter Methylation of BTG3 in the Progression of Hepatocellular Carcinoma
Source: PLoS One. 2013 Oct 17;8(10):e77473. doi: 10.1371/journal.pone.0077473 (PMC3798399; doi:10.1371/journal.pone.0077473)
Supplement: Table S2 — Abbreviations: HR, Hazard radio; CI, Confidence interval. Statistically significant (p<0.05). (DOC) [file pone.0077473.s002.doc]

| Variables | Univariate |  | P value | Multivariate |  | P value |
| --- | --- | --- | --- | --- | --- | --- |
|  | HR | CI (95%) |  | HR | CI (95%) |  |
| BTG3 | 0.238 | 0.058-0.977 | **0.046** | 0.341 | 0.079-1.479 | 0.341 |
| Age | 1.123 | 0.646-1.951 | 0.681 | 1.047 | 0.582-1.883 | 0.879 |
| Gender | 2.18 | 0.787-6.039 | 0.134 | 1.838 | 0.630-5.368 | 0.265 |
| Portal vein thrombosis | 0.469 | 0.258-0.851 | **0.013** | 0.567 | 0.279-1.153 | 0.117 |
| Differentiation | 0.505 | 0.332-0.770 | **0.001** | 0.514 | 0.317-0.833 | **0.007** |
| Cirrhosis | 1.119 | 0.655-1.912 | 0.679 | 0.96 | 0.540-1.707 | 0.888 |
| Tumor size | 1.319 | 0.749-2.192 | 0.366 | 0.871 | 0.474-1.603 | 0.658 |
| Distant metastasis | 0.454 | 0.266-0.774 | **0.004** | 0.953 | 0.499-1.820 | 0.884 |
| Dissemination | 0.4 | 0.225-0.711 | **0.002** | 0.394 | 0.205-0.757 | **0.005** |
| Relapse | 1.887 | 1.102-3.229 | **0.021** | 1.559 | 0.855-2.843 | 0.148 |
| HBsAg | 1.034 | 0.554-1.931 | 0.915 | 1.048 | 0.529-2.076 | 0.894 |
| Serum AFP | 0.883 | 0.513-1.520 | 0.653 | 0.711 | 0.399-1.268 | 0.248 |
