# Supplementary material for: The Suppressive Role and Aberrent Promoter Methylation of BTG3 in the Progression of Hepatocellular Carcinoma
Source: PLoS One. 2013 Oct 17;8(10):e77473. doi: 10.1371/journal.pone.0077473 (PMC3798399; doi:10.1371/journal.pone.0077473)
Supplement: Materials and Methods S1 — (DOC) [file pone.0077473.s003.doc]

**Supporting Information**

**Material and Methods**

***HCC clinical tissue specimens and cell lines***

A total of 141 cases of paraffin specimens of primary HCC were obtained from the Department of Pathology, Nanfang Hospital affiliated with Southern Medical University from 2003 to 2005. Patients included 121 males and 20 females, of ages ranging from 85 to 27 year (mean, 52 years old). They were not pretreated with radiotherapy or chemotherapy prior to surgery. They were followed up for 5 years and their complete clinical data were collected. The histological types were assigned according to the criteria of the World Health Organization (WHO) classification system by three pathologists independently in a double-blinded manner. Twenty matched pairs of fresh HCC tissues were obtained from patients undergoing hepatectomy for liver cancer at Nanfang Hospital from June to October in 2011. Adjacent non-neoplastic tissues from the macroscopic tumor margin (histologically confirmed) were isolated at the same time and used as the control. Informed consent was obtained from all patients prior to tissue acquisition. All the samples were stored in -80°C for RNA analysis and protein expression detection. A hepatocyte cell line (LO2, also named HL-7702) and five HCC cell lines (HepG2, HCCL-M3, MHCC-97H, SMMC-7721 and QGy-7701) were purchased from the cell bank of Chinese Academy of Sciences and all preserved at our laboratory[19-21]. LO2 was maintained in RPMI 1640 (Hyclone, Logan City, UT, USA) supplemented with 20% fetal bovine serum (FBS). Five HCC cell lines were maintained in RPMI 1640 supplemented with 10% FBS.

***Immunohistochemistry (IHC)***

An immunohistochemical assay was performed on 5-mm, formalin-fixed, paraffin-embedded tissue sections according to the following procedures. Briefly, tissue slides were deparaffinized in xylene and rehydrated in graded alcohol. Endogenous peroxidase was blocked with 3% hydrogen peroxide for 10 min, followed by microwave antigen retrieval in 10 mM citrate buffer (pH 6.0). the sections were blocked with 5% normal goat serum for 60 min at room temperature and incubated with a polyclonal, anti-BTG3 antibody (1:100; Abnova, Cambridge, UK) for 60 min at 37°C, After incubation with secondary antibody for 40 min at 37°C, the visualization signal was developed with 3,3’-diaminobenzidine tetrachloride (DAB). The stained tissue sections were reviewed and scored separately by two pathologists blinded to the clinical parameters. The total BTG3 immunostaining score was calculated as the sum of the percent positivity of stained tumor cells and the staining intensity. The percent positivity was scored as ‘‘0,’’ 0%; ‘‘1,’’ 1–25%; ‘‘2,’’ 26–50%; ‘‘3,’’ 51–75% and ‘‘4,’’ >75%. The staining intensity was scored as ‘‘0’’ (no staining), ‘‘1’’ (weakly stained), ‘‘2’’ (moderately stained) and ‘‘3’’ (strongly stained). Both percent positivity of cells and staining intensity were decided in a double blinded manner. The staining of BTG3 was assessed as follows: (−) means a final staining score of <3; (+) a final staining score of 3; (+ +) a final staining score of 4; and (+ + +) a final staining score of ≥5, as shown in Table 1. This relatively simple, reproducible scoring method gives highly concordant results between independent evaluators[1,2]. Cutoff values for BTG3 were chosen based on a measure of heterogeneity by using log-rank statistical analysis with respect to overall survival. An optimal cutoff value was identified: Tumors with a final staining score 0~+ were classified as those with low expression of BTG3, and tumors with a final staining score ++~+++ were classified as those with high expression of BTG3.

***Quantitative real-time polymerase chain reaction (PCR)***

Total cellular RNA was extracted with TRIzol (Invitrogen, Carlsbad, CA, USA) and cDNA was synthesized by using an access reverse transcription system (TaKaRa, Otsu, Japan). The quantitative real-time polymerase chain reaction (PCR) was performed in a total volume of 20 ml, using SYBR Green PCR master mix (TaKaRa, Otsu, Japan). Fluorescence in each well was measured by ABI 7500 Real Time PCR System (Applied Biosystems, Foster City, CA,USA) , according to following thermal cycling profile: 95°C for 30 s, followed by 40 cycles of amplification (95°C for 5 s, 60°C for 34s, 72°C for 30 s). Primer sequences of BTG3 were as follows: forward 5’-TGA AGA AAC AAG TAA GGAA-3’, reverse 5’-TTG GAA GAG GTG GAA ATA-3’. Relative quantity of target transcripts in each sample was expressed as N-fold differences relative to control and according to the equation: 2–DDC. △△Ct = [Ct(target gene) - Ct(GAPDH)]experimental group - [Ct(target gene) - Ct(GAPDH)] control group. Here, the hepatocyte cell line LO2 was regarded as “control group.” Human GAPDH gene amplified as an internal control. Each sample was tested three times and all other quantities were expressed as an n-fold difference relative to the corresponding control group.

***Western blotting***

Cells were washed three times with cold PBS and lysed on ice in RIPA buffer with protease inhibitors, and protein quantification was performed by a BCA protein assay kit (Pierce, Rockford, IL, USA). Equal amounts of proteins (50mg) were electrophoresed on 10% denaturing SDS polyacrylamide gel, and transferred to polyvinylidene fluoride membranes (ImmobilonP; Millipore, Bedford, MA, USA). The membranes were then blocked with 5% non-fat milk and 0.1% Tween20 in Tris-buffered saline, and Membranes were immunoblotted overnight at 4°C with polyclonal anti-BTG3 antibody (Abnova, Cambridge, UK), anti-GAPDH antibody (Santa Cruz Biotechnology, Santa Cruz, USA), anti-cyclin D1 antibody (Abcam, Boston, USA), anti-p27 antibody (Abcam, Boston, USA) followed by their respective secondary antibodies. Signals were detected by enhanced chemiluminescence (Pierce, Rockford, IL, USA).

***Construction of plasmids and transfection***

For overexpression of BTG3, human BTG3 cDNA was amplified by PCR using the following primers: 5'- AGT GCT CGA GAA GAA TGA AAT TGC TGC CGT TGTC-3' and 5'- TGA TCC CGG GTT AGT GAG GTG CTA ACA TGT GAG GAT- 3’. PCR products were cloned into pEGFP-C1 vector. Cells were transfected with BTG3-expressing plasmid by using Lipofectamine 2000 (Invitrogen). After 48h of incubation, RNA and protein of cells were harvested and then used for the evaluation of BTG3 expression. For depletion of BTG3, two human shRNA sequences were cloned into GV102 plasmid, containing EGFP gene and neomycin gene (Genechem, shanghai, China) to generate GV102-BTG3-RNAi(s), respectively; the sequences were shRNA1 (sense, 5’-GGA GCG CAA CAG UUA CCU GTT-3’), shRNA2 (sense, 5’- GGU GAA GGA CUU CCU GAC ATT- 3’) (Invitrogen, Foster City, CA, USA). A scramble siRNA (5’- AAT CGC ATA GCG TAT GCC GTT-3’), which has no homology with the mammalian mRNA sequences, was inserted into GV102 vector as described above. Cells were all transfected with 3 mg of plasmids using Lipofectamine 2000 according to the instructions (Invitrogen). The independent colonies resistant to neomycin were picked up and subcultured in 14 days of selection.

***Statistical analysis***

The expression of BTG3 in HCC tissues compared with adjacent cirrhotic livers or normal livers was analyzed with Wilcoxon Signed Ranks Test. The correlation of BTG3 expression to various clinicopathological parameters was evaluated with chi-suqare test. Survival analyses were performed according to the Kaplan–Meier method and compared by the log-rank test. Cell proliferation, cell cycle and in vitro invasion assays were tested using one-way ANOVA. All statistical analyses were analyzed using the SPSS 13.0 software and P < 0.05 was considered significant.

**References**

1. [Chang CY](http://www.ncbi.nlm.nih.gov/pubmed?term=Chang CY%5BAuthor%5D&cauthor=true&cauthor_uid=21996735), [Lin SC](http://www.ncbi.nlm.nih.gov/pubmed?term=Lin SC%5BAuthor%5D&cauthor=true&cauthor_uid=21996735), [Su WH](http://www.ncbi.nlm.nih.gov/pubmed?term=Su WH%5BAuthor%5D&cauthor=true&cauthor_uid=21996735), et al. Somatic LMCD1 mutations promoted cell migration and tumor metastasis in hepatocellular carcinoma. [Oncogene](http://www.ncbi.nlm.nih.gov/pubmed/21996735) 2012; 31: 2640-2652

2. Huang J, Ye X, Guan J, et al. [Tiam1 is associated with hepatocellular carcinoma metastasis.](http://www.ncbi.nlm.nih.gov/pubmed/22573407) Int J Cancer 2013; 132: 90-100
